# False-Positive Serology for Rocky Mountain Spotted Fever in Long Island, New York, during 2011–2021

**DOI:** 10.3390/pathogens12030503

**Published:** 2023-03-22

**Authors:** Monirul I. Sajib, Pooja Lamba, Eric D. Spitzer, Luis A. Marcos

**Affiliations:** 1 Infectious Disease Division, Department of Medicine, Stony Brook University, Stony Brook, NY 11794, USA; monirul.sajib@stonybrookmedicine.edu (M.I.S.); pooja.lamba@stonybrookmedicine.edu (P.L.); 2 Department of Pathology, Stony Brook University, Stony Brook, NY 11794, USA; eric.spitzer@stonybrookmedicine.edu; 3 Department of Microbiology and Immunology, Stony Brook University, Stony Brook, NY 11794, USA

**Keywords:** rocky mountain spotted fever, serology, tick-borne illness

## Abstract

Cases of rocky mountain spotted fever (RMSF) are increasingly reported every year in Long Island, New York. In clinical practice, an uncommonly high number of referrals with a positive RMSF IgG test result have been seen in our tick-borne disease clinic. The aim of this study is to describe the clinical–epidemiological characteristics and outcomes of hospitalized patients with positive serologies for RMSF in our academic center in Long Island, NY. We found that out of twenty-four patients with a positive serology for RMSF, only one case met the case definition per CDC criteria, two had suspected RMSF, and the other twenty-one did not have a clinical picture consistent with RMSF. A high number of false-positive RMSF serology may be due to other spotted fever rickettsioses in Long Island. Further studies are needed to investigate the presence of another *Rickettsia* spp. (such as *Rickettsia amblyommatis*) in this area that may affect humans.

## 1. Introduction

Front-line clinicians commonly encounter patients with febrile illnesses, and nonspecific flu-like symptoms such as headache, myalgia, malaise, fatigue, sore throat, cough, nausea, and vomiting. In the United States, tick-borne diseases (TBDs) such as ehrlichiosis, anaplasmosis, borreliosis (caused by tick-borne relapsing fever group), babesiosis, and spotted fever rickettsioses (SFR) are often in our differential diagnosis when a patient presents with laboratory abnormalities such as leukopenia, thrombocytopenia, anemia, hyponatremia, abnormal liver, or renal function and has epidemiological risk factors [1,2,3,4]. Among these TBDs, the most potentially lethal one is rocky mountain spotted fever (RMSF), which is an acute tick-borne infection caused by the bacteria *Rickettsia rickettsii*. RMSF can be rapidly fatal within the first five days of the onset of symptoms [5,6]. RMSF may clinically present like the other TBDs initially within the first 3 days of disease. However, the rash from RMSF usually presents 4–5 days after symptom onset, and the infection may result in significant morbidity and mortality if the patient is not diagnosed and treated early [7,8,9]. RMSF is endemic in many parts of the U.S., such as the Southeast, Pacific, and West regions. The tick most commonly associated with the transmission of RMSF is the American dog tick (*Dermacentor variabilis*), and less commonly, the wood tick (*Dermacentor andersoni*) and the brown dog tick, *Rhipicephalus sanguineus* [2]. New York historically has reported RMSF cases [10], including in the city [11], but the cases have increased significantly lately, with a total of 44 reportable cases in 2018. In New York state, the county with the highest number of RMSF cases reported is Suffolk County in Long Island, with 9 reported cases in 2018 [12]. In recent clinical practice, we have anecdotally seen a number of referrals with positive RMSF IgG serology but without a syndrome consistent with RMSF. The annual number of cases of RMSF in the U.S. has increased significantly, from 495 in the year 2000 to more than 5000 cases in 2018 [13]. It is possible that the reactivity in the serology for RMSF may have resulted from other, less severe SFR. Given the high number of RMSF cases seen in Suffolk County, and to better understand factors associated with case definition and outcomes, the aim of this study was to describe the hospitalized cases with a positive RMSF serology in a large academic center located in the epicenter of TBD in Long Island, New York.

## 2. Material and Methods

Study design. A descriptive retrospective study was performed. RMSF serology-positive (indirect immunofluorescence antibody (IFA) assay for IgG or IgM) or positive polymerase chain reaction (PCR) cases (age range: 5–99 years old) were collected at Stony Brook University Hospital (SBUH) from 1 January 2011 to 31 December 2021. Once these cases were identified, a review of their electronic medical records (EMR) was performed.

Inclusion Criteria. Inclusion criteria were to have a patient with a positive RMSF IgM or IgG result, between 5 and 99 years old, who was seen in the SBUH emergency department, or was admitted to the hospital. Outpatient cases were excluded as well as those with no clinical documentation, despite having positive serologies. RMSF IgM and IgG assays (semi-quantitative IFA) were performed at ARUP Laboratories (Salt Lake City, UT, USA).

Clinical–epidemiological data and outcomes. Data collection included demographic characteristics (age and gender), clinical presentation (nausea, vomiting, fever, chills, rash, headache, and joint pain), laboratory data (peripheral white blood cells, hemoglobin, hematocrit, platelets, alanine aminotransferase, aspartate aminotransferase, alkaline phosphatase, total bilirubin, creatinine, and sodium), serological studies for other TBD, and final possible/confirmed diagnosis. Cases were then cross-referenced for the presence of severe disease, which was defined as need for admission to the intensive care unit (ICU).

## 3. Results

A total of 24 patients met the inclusion criteria of having a positive serology for RMSF and clinical documentation of symptoms (a summary is shown in Table 1). The average age was 47.7 years old (range: from 11 to 78 years). Out of the 24 patients, 10 (41.7%) were female and 14 (58.3%) were male. No PCR test for RMSF was available for any of the patients. Only one patient (4.2%) met the CDC case definition for RMSF with a fourfold increase in IgG titers in a convalescent serum sample collected approximately 2 weeks after the initial presentation. Two patients had positive IgM titers of 1:256 and clinical syndromes that were consistent with RMSF. These three patients with RMSF presented between the months of April and August. The other 21 patients had an alternative diagnosis for their presentation, and their RMSF-positive serology was considered either a false-positive result or the result of prior exposure to rickettsial infections. Out of these 21 patients, 3 patients had an IgG titer of 1:128, 1 patient had an IgG titer of 1:256, and 1 patient had an IgG titer of 1:1024; strikingly, 13 patients (54.2%) had a positive IgG titer of 1:64. The remaining three patients’ IgG titers were negative (less than 1:64), but low-level IgM titers (1:64) were detectable.

Although convalescent titers were not available in 23 patients, their initial titers were considered either clinically insignificant, false-positives, or due to past exposure or cross-reactions to other tick-borne diseases by the treating physician. However, for two patients (patients five and six), the treating physician suspected RMSF based on the clinical presentation and serological data, as there was an increase in IgM titers to 1:256. Thus, in this study, there were likely a total of three cases of RMSF, with one admitted to the ICU. There were no fatal outcomes.

Among the remaining 21 patients, 3 patients were subsequently diagnosed with Lyme disease, 2 with ehrlichiosis, 1 with anaplasmosis, and 1 with babesiosis. Ten patients were diagnosed with non-tick-borne-related illnesses such as viral exanthem, acute pyelonephritis, COVID-19 pneumonia, and seronegative rheumatoid arthritis. The diagnosis was unclear in four patients, but their clinical syndrome was not consistent with any TBD.

In 15 of the 24 patients (62.5%), the RMSF serologies were obtained during warm months (between April and September), whereas in the other 9 patients (37.5%), the serologies were obtained during the colder months (November and March).

## 4. Discussion

We found a number of likely false-positive cases of RMSF serology, single-time point-positives, in this endemic area of New York, which may have implications for clinical diagnostic interpretation. Positive serologies for RMSF can result from infection with spotted fever group rickettsiae (SFGR), which includes *R. rickettsii*, as well as other pathogens such as *R. parkerii*; this may explain the increases in SFR in the U.S. from 2010 to 2018 [14]. *R. amblyommatis,* which is related to members of the SFGR, is widely present in the lone star tick, *Amblyomma americanum*. Up to 61% of adult lone star ticks, the predominant tick in Long Island, New York, harbor *R. amblyommatis* [15]. *A. americanum* is well known for being the vector that transmits the disease ehrlichiosis [16]. In contrast, the pathogenicity of *R. amblyommatis* for humans is unknown. Interestingly, the number of cases of SFR reported in the U.S. has substantially increased during the past several decades, but the fatality rate has decreased during the same period of time [17]. Modeling studies evaluating the relationship between SFG rickettsiosis and changes in the presence of *A. americanum* populations suggest that the increases in reported cases of SFR are associated with the expansion of the geographic range of *A. americanum* in the U.S. [17]. Geographic differences in SFG serology results, potentially related to the prevalence of *A. americanum*, were noted by Starily et al., who observed that 11.1% of blood donors in Georgia were positive for *R. rickettsii* antibodies with titers ≥ 64, whereas only 6.3% of Oregon/Washington donors had *R. rickettsii* titers of ≥64; of these positive sera, 64% had a titer of 64 and only 4% had a titer ≥ 256 [18]. In a study from Tennessee, Delisle et al. observed that in a collection of 56 SFG-positive specimens from a commercial reference laboratory, 80–90% of the samples were cross-reactive with *R. rickettsii, R. parkeri, R. montanensis,* and *R. amblyommatis* antigens, but after cross-absorption of the sera, 55% of the samples showed specific reactivity with *R. amblyommatis* antigens while none showed specific reactivity with *R. ricketsii* (45% showed indeterminate specificities) [19]. Whether *R. amblyommatis* or another *Rickettsia* spp. may be responsible for this low-titer-positive serology for RMSF is still an open question in Long Island. However, the hypothesis that some of the increase in cases positive for RMSF in NY is secondary to *R. amblyommatis* exposure and/or infection is consistent with increases in the prevalence of *A. americanum* in Long Island during the past two decades [20].

Mortality rates in RMSF are high, and serology may be negative in acute infections [21] when the period of infection is less than 5–7 days. This study was performed in hospitalized patients with RMSF-positive serology because these complicated cases would likely have been infected for more than 5 days upon arrival to the emergency department. By this stage, the IgM and/or IgG antibodies may start to rise. We detected one confirmed case and two possible cases of RMSF during this 11-year retrospective study.

In our series of cases, three patients had only a single low-titer-positive IgM for RMSF, but their clinical presentation was not consistent with RMSF. IgM RMSF-positive results should be interpreted cautiously. A positive IgM for RMSF may lead to an improper classification of a patient as a possible RMSF case when indeed, it may be only a false-positive test [22]. There may be several reasons for these false-positive RMSF results. We found that in the vast majority of our cases, RMSF serology was performed along with other diagnostic tests for TBD that are endemic in this region. The initial clinical presentation of these cases was a flu-like syndrome, which was sometimes associated with initial laboratory findings such as leukopenia, thrombocytopenia, and transaminitis (as shown in Table 1). For instance, patients #1, 5, and 6 were empirically treated with doxycycline due to a high clinical suspicion for RMSF. In addition, patients #2, 3, 4, 7, 11, and 12 were also treated with doxycycline for either suspected or confirmed TBD. Patient #16 was treated with ceftriaxone for Lyme disease (neuroborreliosis). In other patients, doxycycline was not prescribed because the initial diagnosis was a viral infection and not TBD. Lastly, given that most patients did not have a clinical presentation that was consistent with RMSF at the end of hospitalization, convalescent titers were not performed.

There are a few limitations to our study. Firstly, the sample size is small, and it is limited to a single academic medical center, which may not be applicable to other patient populations. Second, the lack of RMSF convalescent serology in many patients and the RMSF PCR (however, he low sensitivity of this assay may have underdiagnosed some of the cases) may result in a missed opportunity to definitely rule out a possible diagnosis of RMSF. A further study looking for changing antibody titers would be useful to confirm a diagnosis of RMSF and other SFGR. Lastly, it would be useful to have commercial testing for *R. amblyommatis.*

For RMSF, a four-fold rise in serum antibodies provides the best evidence for current infection; however, it seems that physicians often rely on single serum samples to evaluate suspected RMSF [23]. Convalescent specimens to confirm infections are required for RMSF, but according to this study, it seems that only 1 out of 24 patients had convalescent titers done in his follow-up.

In conclusion, the number of RMSF cases remains low in our academic center. The relatively large number of positive RMSF serology cases (IgG titer ≥ 1:64) found in this study may be related to cross-reactions with *R. amblyommatis*, which is endemic in lone star ticks in Long Island. We suspect that *R. amblyommatis* may play a role in causing mild febrile flu-like symptoms during the warmer season (spring to fall) in our area. Further studies are needed to clarify this hypothesis.

## Figures and Tables

**Table 1:** Clinical–Epidemiological Characteristics of RMSF serologically positive cases seen at SBUH 2011–2021.

Patient #	Age (Years)	Gender	RMSF Serology (Titers)	Pertinent Symptoms/Relevant History (Month/Year of Presentation)	Pertinent Labs on Initial Presentation (WBC: K/uL, Hg: g/dL, Platelets: K/uL, AST: IU/L, and ALT: IU/L)	Significant Diagnosis/Clinical Course
1 *	15	M	IgM: 1:128; IgG: 1:64 Convalescent: IgM: 1:256; IgG: 1:256	Fever, myalgia, headache, and rash (August 2012)	WBC: 4.8, Hg: 11.3, platelet: 62, AST: 115, and ALT: 74	RMSF and required ICU admission
2	76	M	IgM: < 1:64 IgG: 1:64	Fever, headache, and rash (November 2021)	WBC: 6.29, Hg: 13.9, platelet: 123, AST: 23, and ALT: 18	Possible viral exanthema
3	38	M	IgM: < 1:64 IgG: 1:64	Rash (June 2020)	WBC: 9.73, Hg: 11.2, platelet: 140, AST: 110, and ALT: 39	Lyme disease (IgG: 5 bands; IgM: 1 band)
4	35	M	IgM: < 1:64 IgG: 1:64	Fever, myalgia, headache, diarrhea, and nausea (July 2020)	WBC: 4.29, Hg: 17, platelet: 73, AST: 95, and ALT: 61	Ehrlichiosis
5 *	14	F	IgM: 1:256 IgG: 1:128	Fever, headache, rash, and history of preceding tick bite ** (May 2012)	WBC: 6.6, Hg: 13.1, platelet: 179, AST: 29, and ALT: 40	Suspected RMSF
6 *	11	M	IgM < 1:64, IgG < 1:64 (2 days later: IgM: 1:256; IgG: < 1:64)	Fever, rash, confusion, nausea, and vomiting (April 2012)	WBC: 5.4, Hg: 11.5, platelet: 142, AST: 138, and ALT: 273	Suspected RMSF
7	57	M	IgM: < 1:64 IgG: 1:1024	Fever, nausea, vomiting, abdominal pain, and diarrhea (August 2020)	WBC: 14.4, Hg: 13.2, platelet: >124, AST: 44, and ALT: 57	Ehrlichiosis and viral gastroenteritis
8	74	F	IgM: < 1:64 IgG: 1:128	Fever, cough, dyspnea, and fatigue (November 2021)	WBC: 8.3, Hg: 10.9, platelet: 270, AST: 19, and ALT: 29	COVID-19 pneumonia
9	52	F	IgM: 1:128 IgG: < 1:64 (2 months later: IgM: 1:256; IgG: < 1:64)	Pain and paresthesia in right hand and fingers (February 2021)	WBC: 6.5, Hg: 13.3, platelet: 243, AST: 19, and ALT: 13	Seronegative rheumatoid arthritis
10	59	M	IgM: < 1:64 IgG: 1:128	Left hip pain, recent tick bite (lone star, deer tick), and history of RMSF (June 2021)	WBC: 6.7, Hg: 14.2, platelet: 177, AST: 20, and ALT: 15	Hip strain/bursitis
11	63	F	IgM: < 1:64 IgG: 1:64	Fever, chills, nausea, vomiting, and malaise (June 2020)	WBC: 2.2, Hg: 12.6, platelet: 28, AST: 74, and ALT: 59	Anaplasmosis
12	66	M	IgM: < 1:64 IgG: 1:64	Fever, headache, and history of tick bite ** (August 2021)	N/A	Lyme disease
13	73	M	IgM: < 1:64 IgG: 1:256	Fever, rigor, diaphoresis, fatigue, and confusion (November 2021)	WBC: 5.5, Hg: 13.4, platelet: 39, AST: 137, and ALT: 142	Babesiosis
14	37	F	IgM: < 1:64 IgG: 1:64	Fatigue, joint pain, and history of tick bite ** (March 2020)	WBC: 6.3, Hg: 12.9, platelet: 340, AST: 25, and ALT: 26	Unclear diagnosis
15	67	M	IgM: < 1:64 IgG: 1:64	Skin lesion/ulcer and history of possible insect and/or tick bite ** (January 2021)	WBC: 7.1, Hg: 16.8, and platelet: 160	Cellulitis
16	56	M	IgM: < 1:64 IgG: 1:64	Paresthesia in bilateral feet and history of Lyme disease (February 2020)	N/A	Lyme disease
17	78	F	IgM: < 1:64 IgG: 1:64	Fever, malaise, nausea, vomiting, diarrhea, and cough (June 2020)	WBC: 3.7, Hg: 12.0, platelet: 125, AST: 27, and ALT: 15	COVID-19 pneumonia
18	39	M	IgM: < 1:64 IgG: 1:64	Headache, myalgia, photophobia, phonophobia, paresthesia in bilateral hands, landscaper, and reported history of tick-borne illness (July 2021)	WBC: 8.4, Hg: 14.4, platelet: 269, AST: 21, and ALT: 28	Unclear diagnosis [post-COVID-19 syndrome suspected]
19	17	F	IgM: < 1:64 IgG: 1:64	Fever, left flank pain, nausea, vomiting, and history of remote tick bite ** (August 2020)	WBC: 13.2, Hg: 13.3, platelet: 239, AST: 14, and ALT: 11	Left pyelonephritis
20	62	F	IgM: < 1:64 IgG: 1:64	Fatigue, intermittent myalgia and paresthesia, arthralgia, and history of tick bite ** (September 2021)	N/A	Unclear diagnosis (other tick-borne work ups negative)
21	38	F	IgM: 1:64 IgG: < 1:64	Chronic fatigue, restlessness, and history of Hashimoto thyroiditis (March 2021)	WBC: 6.01, Hg: 11.3, platelet: 262, AST: 16, and ALT: 7	Suspected autoimmune/connective tissue disease
22	37	M	IgM: 1:64 IgG: < 1:64	Fever, dizziness, neck, and back pain (September 2011)	WBC: 5.4, Hg: 14.8, platelet: 210, AST: 34, and ALT: 41	Unclear diagnosis
23	40	M	IgM: < 1:64 IgG: 1:128	Fever, headache, and lethargy (February 2012)	WBC: 11.6, Hg: 14.9, platelet: 121, AST: 72, and ALT: 102	Viral syndrome
24	40	F	IgM: < 1:64 IgG: 1:64	Fever, chills, rash, headache, myalgia, migratory joint pain, neck pain, photophobia, and history of tick bite ** (August 2011)	WBC: 7.2, Hg: 10.5, platelet: 208, AST: 22, and ALT: 11	Viral illness

M: male; F: female; WBC: white blood cell count (units cells/mL); Hg (units dg/L); platelets (units/mL); AST: alanine aminotransferase; AST: aspartate aminotransferase. N/A: Not available. ** Some patients reported a history of tick bite (species unknown). * RMSF cases. # = number of patients.

## Data Availability

Data is contained within the article.
